# Fabrication and characterization of ZnO/Se_1-*x*_Te_*x*_ solar cells

**DOI:** 10.1007/s12200-022-00040-5

**Published:** 2022-09-08

**Authors:** Jiajia Zheng, Liuchong Fu, Yuming He, Kanghua Li, Yue Lu, Jiayou Xue, Yuxuan Liu, Chong Dong, Chao Chen, Jiang Tang

**Affiliations:** 1 Wuhan National Laboratory for Optoelectronics (WNLO) and School of Optical and Electronic Information, Huazhong University of Science and Technology, Wuhan, 430074 China; 2 China-EU Institute for Clean and Renewable Energy (ICARE), Huazhong University of Science and Technology, Wuhan, 430074 China

**Keywords:** Se_1−*x*_Te_*x*_ alloy, ZnO electron transport layer, Recombination mechanism, Solar cells

## Abstract

Selenium (Se) element is a promising light-harvesting material for solar cells because of the large absorption coefficient and prominent photoconductivity. However, the efficiency of Se solar cells has been stagnated for a long time owing to the suboptimal bandgap (> 1.8 eV) and the lack of a proper electron transport layer. In this work, we tune the bandgap of the absorber to the optimal value of Shockley–Queisser limit (1.36 eV) by alloying 30% Te with 70% Se. Simultaneously, ZnO electron transport layer is selected because of the proper band alignment, and the mild reaction at ZnO/Se_0.7_Te_0.3_ interface guarantees a good-quality heterojunction. Finally, a superior efficiency of 1.85% is achieved on ZnO/Se_0.7_Te_0.3_ solar cells.

**Graphical abstract:**

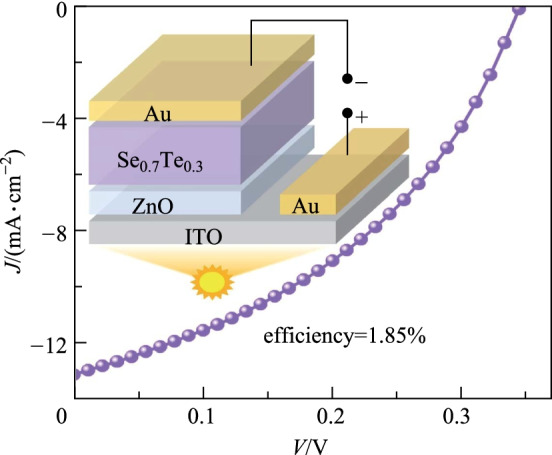

**Supplementary Information:**

The online version contains supplementary material available at 10.1007/s12200-022-00040-5.

## Introduction

Selenium (Se) is the oldest photovoltaic (PV) semiconductor material [1] with a bandgap (*E*_g_) of 1.8–2.0 eV [2, 3], a large absorption coefficient (> 10^4^ cm^−1^) at visible spectrum [4], and a theoretical Shockley–Queisser (S–Q) limit beyond 20% [5, 6]. Compared with Cu_2_(In,Ga)(S,Se)_2_ [7], organic–inorganic hybrid halide perovskites [8–10] and CdTe [11] photovoltaic materials, Se has the advantages of easy phase control, good air stability and nontoxicity. Meanwhile, benefitting from the low melting point (221 °C) and high vapor pressure (1.1 Pa @250 °C) [12], Se film can be prepared at low temperature by vacuum technologies on various substrates [13–17], showcasing a great potential for large-scale production.

Se solar cells, based on indium tin oxide (ITO)/TiO_2_/Se/Au device structure, reached an impressive efficiency of 5% in 1985 [15], but the progress was slow for the following 30 years [16, 18–20]. Si advanced so rapidly that Se has not received much attention for a long time [21, 22]. Until 2017, Todorov et al. created a record efficiency of 6.5% by optimizing the functional layer thickness and adopting a MoO_*x*_ hole transport layer [17]. It is notable that the bandgap of Se is out of the optimal range of S–Q limit (1–1.5 eV), so it would lead to an inadequate use of sunlight and thereby a low photocurrent. Tellurium (Te), as the congener of Se, has a narrow bandgap of 0.33 eV [23] and the same hexagonal crystal structure [24], thus, it is possible to continuously tune the bandgap of Se_1−*x*_Te_*x*_ to the optimal S–Q bandgap of 1.36 eV. Se and Te are two less-studied photovoltaic materials and stand out for their simple composition, high carrier mobility, good air stability, high photoconductivity and thermoelectric response [25, 26]. They are also the significant components of transition metal dichalcogenides (TMDCs), which are widely applied in high-performance field-effect transistors (FETs) [27, 28], optoelectronic devices [29], and thermoelectric devices.

Owing to the tunable photoconductivity and optical response of Se_1−*x*_Te_*x*_, it has been used for solar cells [30], short-wave infrared photodetectors [31] and semiconductor core optical fibers [32]. In 2019, Hadar et al*.* investigated Se_1−*x*_Te_*x*_ films for PV application [30], but the efficiency of the alloy solar cells is less than 3%, only half of the pure Se solar cells. Therefore, it is important to choose the Se_1−*x*_Te_*x*_ film with a suitable component and bandgap. In addition, the current Se and Se_1−*x*_Te_*x*_ solar cells commonly adopt TiO_2_ as an electron transport layer (ETL) [15, 16, 20]. Unfortunately the inertness of TiO_2_ surface cannot be bonded with Se_1−*x*_Te_*x*_ tightly and may potentially give rise to an inferior interface with poor adhesion. ZnO surface is more reactive than TiO_2_, and ZnO has a higher electron mobility (> 150 cm^2^/(V·s)) [33] and lower fabrication temperature [34]. Therefore, ZnO is a preferred alternative compared to TiO_2_.

In this work, we optimized the component and bandgap of Se_1−*x*_Te_*x*_ absorber and adopted the active ZnO ETL to assemble solar cell devices. First, we alloyed Se films with Te at certain molar ratios (*x* = [Te] = 0.2, 0.3, 0.4, 0.5) and tuned the bandgap from 1.53 to 1.13 eV. Based on the S–Q limit, we chose the Se_0.7_Te_0.3_ film with a bandgap of 1.36 eV for the target absorber material. Then, combining the band alignment and surface reactivity, ZnO ETL was selected to construct ITO/ZnO/Se_1−*x*_Te_*x*_/Au solar cells. Factually, theoretical thermodynamic calculation confirmed that ZnO can react with Se, and the Zn^2+^ exposed at (111) polar surface of ZnO fabricated by magnetron sputtering under oxygen-poor condition (O:Ar = 1:99) is more conducive to the formation of Zn-Se bonds at the ZnO/Se interface. Therefore, it can help to form a strong-adhesion interface and obtain satisfactory device performance. Finally, we achieved a superior efficiency of 1.85% on ITO/ZnO/Se_0.7_Te_0.3_/Au solar cell.

## Experimental section

### Film and device preparation

For the preparation of Se_1−*x*_Te_*x*_ raw materials, a certain proportion (*x* = [Te] = 0.2, 0.3, 0.4, 0.5) of Se and Te powder (99.999% purity, Aladdin) were sealed in a quartz tube, then heated at 560 °C in a muffle furnace for 24 h, and slowly cooled to room temperature with a cooling rate of 22 °C/h. For device preparation, the ITO glass (Kaivo, Zhuhai, China) with the square resistance of 6–8 Ω/sq was used as the substrate. The ITO substrates had been cleaned using a detergent, isopropanol, ethyl alcohol and DI water rinsing in sequence. Then 1 μm Se_1−*x*_Te_*x*_ films were deposited by thermal evaporation (Kurt J. Lesker, ~ 5 × 10^−3^ Pa), and annealed at 200 °C for 2 min on a heating stage in the glove box. Subsequently, ZnO films (180 nm thickness) were prepared by magnetron sputtering (JCP500, Technol Science; O:Ar = 1:99 atmosphere). Finally, Au electrodes (0.09 cm^2^ area, 100 nm thickness) were evaporated by the resistance evaporation thin-film system (Beijing Technol Science) under a vacuum pressure of 5 × 10^−3^ Pa.

### Film characterization

The morphologies and energy dispersive spectroscopy (EDS) characterization of Se_0.7_Te_0.3_ films were checked by scanning electron microscopy (SEM, GeminiSEM, Zeiss, without Pt coating). The X-ray diffraction (XRD) with Cu Kα radiation (Empyrean, PANalytical B.V.) was carried out to determine the component and orientation of Se_0.7_Te_0.3_ and ZnO films. The morphologies of the Se_1−*x*_Te_*x*_ and ZnO films were observed by the atomic force microscope (AFM, SPM9700, Shimadzu). The optical transmittance of Se_1−*x*_Te_*x*_ film was recorded by UV–Vis spectrophotometer (PerkinElmer Instruments, Lambda 950 using integrating sphere). Ultraviolet photoelectron spectroscopy (UPS, AXIS-ULTRA DLD-600 W, Kratos) was used to confirm the energy level positions of Se_0.7_Te_0.3_. The Hall coefficient and carrier concentration were obtained via a Hall measurement system (Ecopia HMS5500). The X-ray photoelectron spectroscopy (XPS, AXIS-ULTRA DLD-600 W) was used to characterize the interface between Se_1−*x*_Te_*x*_ and ZnO).

### Device characterization

The device performance was characterized by a digital source meter (Keithley2400) under simulated AM 1.5G solar (Oriel 94023A, light intensity of 1000 mW/cm^2^ calibrated with a standard silicon cell). external quantum efficiency (EQE) measurements were carried out using a 300 W xenon lamp of Newport (Oriel, 69911) as a light source and a Newport oriel cornerstone TM 130 1/8 Monochromator (Oriel, model 74004) to split light into monochromatic waves. Capacitance–voltage (*C–V*) and drive-level capacitance profiling (DLCP) measurement was carried out with Keithley 4200-CVU module at a frequency of 70 kHz. Temperature dependent admittance spectral (AS, Agilent E4980A LCR meter) was used for temperature-dependent AS and conductivity measurements, and samples were put in a liquid nitrogen cryostat (Janis VPF-100). The temperature was controlled by a temperature controller (Lakeshore 325) and ranged from 80 to 320 K at a step of 10 K. When the setting temperature was stable, AS and current–voltage (*I*–*V*) measurements were performed using an impedance analyzer (Agilent E4980A LCR meter) and a semiconductor device parameter analyzer (Agilent B1500A), respectively.

## Results and discussion

**Fig. 1:**
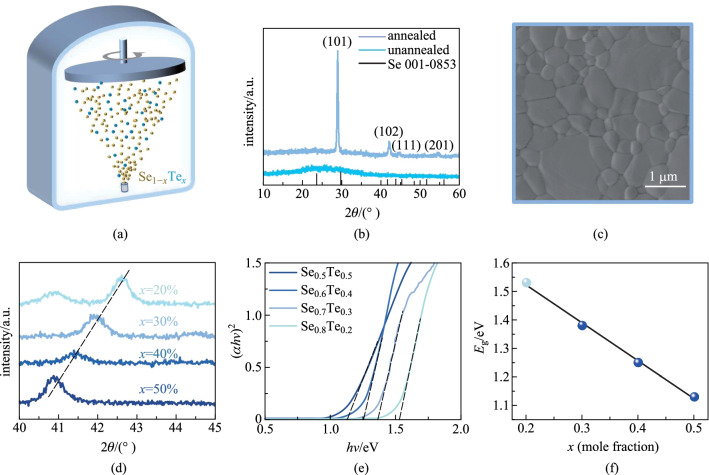
**a** Schematic diagram of thermal evaporation. **b** XRD of Se_0.7_Te_0.3_ film before and after annealing. **c** SEM image of the annealed Se_0.7_Te_0.3_ film. **d** (102) diffraction peaks of Se_1−*x*_Te_*x*_ films. **e** Bandgaps of alloying films. **f** Bandgaps of Se_1−*x*_Te_*x*_ films as a function of *x*

A certain proportion of Se and Te powder were mixed evenly to form Se_1−*x*_Te_*x*_ (*x* = 0.2, 0.3, 0.4, 0.5) blocks (Additional file 1: Fig. S1). Then the Se_1−*x*_Te_*x*_ films were deposited at room temperature by thermal evaporation (Fig. 1a) using Se_1−*x*_Te_*x*_ powder ground from the blocks. The as-deposited films were amorphous (Additional file 1: Fig. S2a), so a post-annealing process was required. The film with intermediate component *x* = [Te] = 0.3 was selected to study the annealing temperature from 150 to 250 °C. The film annealed at 250 °C for 2 min was thermally decomposed because of the high saturated vapor pressure at this temperature (Additional file 1: Fig. S3d), and the film annealed at 150 °C for 2 min is incompletely crystallized (Additional file 1: Fig. S3a). When the annealing temperature was 200 °C for 2 min, the film showed flat surface, densely arranged grains and high crystallinity (Fig. 1b, c), which meets the requirements of high-efficiency solar cells. Allowing for the high vapor pressure of Se, Se may escape from Se_1−*x*_Te_*x*_ films during the annealing process, giving rise to the deviation from the target component. The EDS indicated that the measured Se:(Se + Te) composition of Se_0.7_Te_0.3_ film is 0.699 (Additional file 1: Fig. S4), consistent with the feeding ratio of 0.7. Therefore, the annealing process is reasonable. Subsequently, all Se_1−*x*_Te_*x*_ films were crystallized at 200 °C for 2 min.

The XRD patterns of annealed Se_1−*x*_Te_*x*_ films with 2*θ* ranging from 10° to 60° are shown in Additional file 1: Fig. S2b, and the zoom-in diffraction peak of (102) is depicted in Fig. 1d. The (102) peaks shift to a small degree with the increase of *x* in accordance with the Bragg’s Law [35] and the content of Te was calculated as expected (Additional file 1: Table S1). The morphologies of the Se_1−*x*_Te_*x*_ films before and after annealing were observed by AFM, which exhibits larger grains and stronger crystallinity after annealing (Additional file 1: Fig. S5). With the increase of Te content, both the grain size of Se_1−*x*_Te_*x*_ films, and the full width at half maxima (FWHM) of (102) peak gradually decrease (Additional file 1: Fig. S5 and Table S1). This indicates a decrease in crystallinity. The transmittance and reflectance spectra were measured on an UV–Vis spectrophotometer to determine the bandgaps of the crystallized Se_1−*x*_Te_*x*_ films (Additional file 1: Fig. S6). Using Tauc method [36], the bandgaps of Se_1−*x*_Te_*x*_ films with *x* = 0.2, 0.3, 0.4, 0.5 are fitted as 1.53, 1.36, 1.25 and 1.13 eV, respectively (Fig. 1e). The bandgap has a linear relationship with *x* (Fig. 1f), which well satisfies the Vegard’s law (Eq. (1)) [37],

1 $${E}_{\mathrm{g}\left({\mathrm{Se}}_{1-x}{\mathrm{Te}}_{x}\right)}=\left(1-x\right){E}_{\mathrm{g}\left(\mathrm{Se}\right)}+x{E}_{\mathrm{g}(\mathrm{Te})},$$

where *E*_g(Se)_ = 1.83 eV is the bandgap of Se, and *E*_g(Te)_ = 0.33 eV is the bandgap of Te. Among them, Se_0.7_Te_0.3_ film with a bandgap of 1.36 eV has more potential according to the S−Q limit.

The position of energy levels, conduction type and carrier density are important to design the solar cell structure. Ultraviolet photoelectron spectroscopy (UPS) of Se_0.7_Te_0.3_ film demonstrated that the valence band maximum (VBM) and conduction band minimum (CBM) of the annealed Se_0.7_Te_0.3_ film are − 5.31 and − 3.95 eV, respectively (Additional file 1: Fig. S7). The detailed calculation process to obtain the VBM and CBM is shown in Additional file 1:. The positive Hall coefficient (*R*_H_, Additional file 1: Table S2) further confirmed that the Se_0.7_Te_0.3_ film is p-type.

An n-type ELT is needed to construct a heterojunction with p-type Se_0.7_Te_0.3_ film. Here, we selected the n-type ZnO because of its higher electron mobility and lower synthesis temperature than the commonly used TiO_2_. Gibbs free energy calculation (Eq. (2), Table 1) [38] shows ZnO can slightly react with Se_1−*x*_Te_*x*_ during 200 °C annealing, but TiO cannot. ZnO and TiO_2_ were further compared experimentally and ZnO showed a better performance as shown in Additional file 1: Fig. S8.

**Table 1:** ${\Delta }_{\mathrm{r}}{G}_{\mathrm{m}}^{\ominus }$ of reactions between Se and ZnO (TiO_2_) at 200 °C annealing temperature

Reaction formula	${\Delta }_{r}{G}_{\mathrm{m}}^{\ominus }\left(T\right)$/(kJ·mol^−1^)
$3\mathrm{Se }\left(\mathrm{g}\right)+2\mathrm{ZnO }\left(\mathrm{s}\right)\stackrel{200\,\space ^\circ{\rm C} }{\longrightarrow }2\mathrm{ZnSe }\left(\mathrm{s}\right)+{\mathrm{SeO}}_{2}\left(\mathrm{s}\right)$	− 455.78
$3\mathrm{Se }\left(\mathrm{g}\right)+{\mathrm{TiO}}_{2} \left(\mathrm{s}\right)\stackrel{200\,\space ^\circ{\rm C} }{\longrightarrow }2{\mathrm{TiSe}}_{2} \left(\mathrm{s}\right)+{\mathrm{SeO}}_{2}\left(\mathrm{s}\right)$	138.82

2 $${\Delta }_{\mathrm{r}}{G}_{\mathrm{m}}^{\ominus }\left(T\right)={\Delta }_{\mathrm{r}}{H}_{\mathrm{m}}^{\ominus }\left(25\,\space\mathrm{^\circ{\rm C} }\right)-T{{\Delta }_{\mathrm{r}}S}_{\mathrm{m}}^{\ominus }\left(25\,\space\mathrm{^\circ{\rm C} }\right),$$

where ${\Delta }_{\mathrm{r}}{G}_{\mathrm{m}}^{\ominus }$, ${\Delta }_{\mathrm{r}}{H}_{\mathrm{m}}^{\ominus }$ and ${{\Delta }_{\mathrm{r}}S}_{\mathrm{m}}^{\ominus }$ mean the changes of Gibbs free energy, enthalpy and entropy, respectively, and *T* is the temperature. The parameters and results of the calculation procedure are shown in Additional file 1: Tables S3 and S4 [39–41]. The existence of ZnSe is proven by the XPS measurement (Additional file 1: Fig. S9d, e). The ZnSe transition layer can enhance the adhesion between Se_1−*x*_Te_*x*_ and ZnO substrate and benefit the low-defectivity ZnO/Se_1−*x*_Te_*x*_ heterojunction interface (see Additional file 1 for experimental details). The finally designed device structure is shown in Fig. 2a, where ITO and gold with high work function are chosen as front and back electrodes, respectively.

**Fig. 2:**
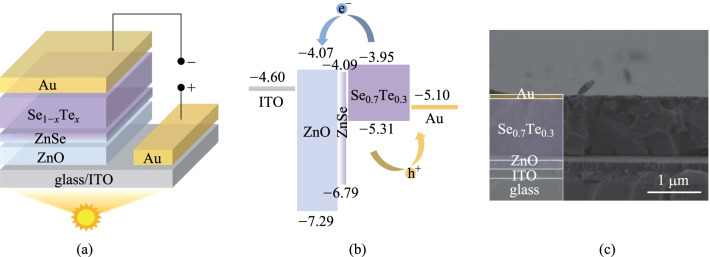
**a** Structure diagram of the Se_0.7_Te_0.3_ solar cells. **b** Band alignment diagram and **c** cross-section SEM image of ITO/ZnO/Se_0.7_Te_0.3_/Au solar cells

ZnO prepared by magnetron sputtering, shows a wide bandgap of 3.22 eV as depicted in Additional file 1: Fig. S10a, thus it does not limit the absorption efficiency of the absorber at visible band. In addition, the smooth, uniform and compact surface of ZnO (1.712 nm roughness and ~ 80 nm grain size, Additional file 1: Fig. S11) is conducive to the subsequent fabrication of Se_1−*x*_Te_*x*_ absorbers and gold electrodes (see Additional file 1 for detailed descriptions). The XRD of ZnO films shows that the preferred orientation is polar (111) facet (Additional file 1: Fig. S10b). According to the first-principle calculation [42], the Zn-terminal (111) facet has lower energy than the O-terminal facet. Therefore, our ZnO film is conducive to the formation of a thin Zn-Se transition layer at the interface with Se_1−*x*_Te_*x*_ film. Combining the energy band of ZnO [43], ZnSe [44], and Se_0.7_Te_0.3_, the band alignment is shown in Fig. 2b, which demonstrates no transport barrier for photogenerated carriers. The cross-section SEM image of the device (Fig. 2c) displays a decent interface. The thickness of ZnO and Se_1−*x*_Te_*x*_ were 180 and 1000 nm, respectively, but the expected ZnSe was too thin to be observed by cross-section SEM.

The device performance was characterized by a digital source meter under simulated AM 1.5G solar. The current density–voltage (*J*–*V*) curves of Se_1−*x*_Te_*x*_ solar cells are depicted in Additional file 1: Fig. S12a and the average and errors of efficiency are depicted in Additional file 1: Fig. S13a. As shown in Table 2, with *x* increases, the open-circuit voltage (*V*_OC_) of Se_1−*x*_Te_*x*_ solar cells decreases as expected, but the short-circuit current (*J*_SC_) does not always increase due to the current loss at long wavelengths according to Additional file 1: Fig. S12c. In addition, the fill factor (FF) of Se_1−*x*_Te_*x*_ solar cells is rather low because of the cliff at the interface and the leakage according to the small shunt resistance (*R*_sh_) as shown in Additional file 1: Table S5. Then Se_1−*x*_Te_*x*_ solar cells with *x* = 0.2, 0.3, 0.4 and 0.5 showed efficiencies of 0.76%, 0.81%, 0.67% and 0.72%, respectively (Table 2). Among them, Se_0.7_Te_0.3_ solar cell stood out with a better balance between *V*_OC_ and *J*_SC_. Thus, we mainly focused on the Se_0.7_Te_0.3_ device and analyzed its air stability, defect properties and recombination mechanism, for the sake of providing guidance for the further performance optimization.

**Table 2:** Device performance parameters of ZnO/Se_1−*x*_Te_*x*_ (*x* = 0.2, 0.3, 0.4, 0.5) solar cells

*x*	*V*_OC_/V	*J*_SC_/(mA·cm^−2^)	FF/%	Efficiency/%
0	0.620	8.1	33.0	1.66
0.2	0.323	6.3	37.4	0.76
0.3	0.255	8.5	37.4	0.81
0.4	0.242	7.7	35.9	0.67
0.5	0.220	9.6	33.6	0.72

**Fig. 3:**
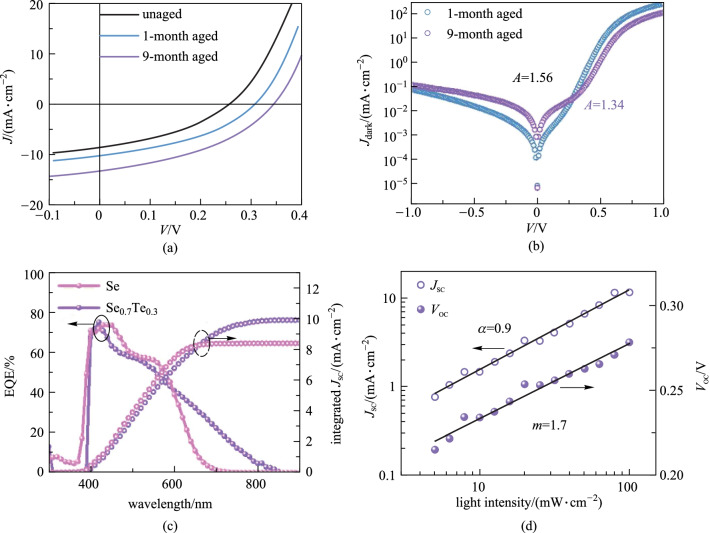
**a** *J*–*V* curves of ZnO/Se_0.7_Te_0.3_ solar cells for different aging time under AM 1.5G solar illumination. **b** Dark *J–V* curves of ZnO/Se_0.7_Te_0.3_ solar cells after 1-month and 9-month aging. **c** EQE spectra of Se_0.7_Te_0.3_ and pure Se solar cells. **d** *J*_SC_ and *V*_OC_ curves as a function of light intensity

For the air stability, we found that the unencapsulated Se_0.7_Te_0.3_ solar cells demonstrated an improved efficiency from 0.81% to 1.25% after 1-month storage in ambient conditions (Fig. 3a and Table 3), as well as the other Se_1−*x*_Te_*x*_ device (Additional file 1: Figs. S12b, S13b and Table S5). After 9 months, the efficiency of Se_0.7_Te_0.3_ device further increased to 1.85% (Fig. 3a and Table 3), a similar phenomenon was also observed by Todorov et al. [17]

**Table 3:** Device performance parameters of ZnO/Se_0.7_Te_0.3_ solar cells for different aging times

Condition	*V*_OC_ /V	*J*_SC_ /(mA·cm^−2^)	FF /%	Efficiency /%	*R*_s_ /Ω	*R*_sh_ /Ω
Fresh	0.255	8.5	37.4	0.81	149	940
1-month aged	0.307	10.2	40.0	1.25	124	882
9-month aged	0.345	13.1	40.9	1.85	99	849

To analyze the degree of defect recombination of aged Se_0.7_Te_0.3_ devices, the quality factor (*A*) fitting [45] and Hall effect measurement were conducted. By fitting the dark *J*–*V* curve [5, 46, 47], the *A* (1.34–1.41) of Se_0.7_Te_0.3_ device, after 9-month aging was obtained, smaller than that (1.56) after 1-month aging (Fig. 3b). Through Hall effect measurement, the carrier concentration (*p*) of Se_0.7_Te_0.3_ film after 1-month was 1.88 × 10^14^ cm^−3^ (Additional file 1: Table S2), while the *p* after 9-month aging was too small to be measured. The smaller *A* and Hall effect results illustrate the lower defect recombination (see Additional file 1 for more analysis about *A*). The mechanism of defect reduction in Se_0.7_Te_0.3_ film can be explained by the low diffusion barrier (0.16 eV) of Se (or Te) vacancy along Se–Se (or Te–Te) chains as shown in Additional file 1: Fig. S14 [48]. It means that the defects in Se_0.7_Te_0.3_ can reduce by the way of a self-healing process, resulting in better device performance.

Although Se_0.7_Te_0.3_ has great potential compared to Se, the device performance is inferior to the pure Se solar cells at the current stage. Inspired by Cao’s work [49], a multi-junction Se_1−*x*_Te_*x*_-based solar cell, with the gradient distribution of the absorbers in each sub-cell to absorb the full solar spectrum, will optimize the efficiency in the future. But for now, we are focusing on the performance improvements of Se_1−*x*_Te_*x*_ single-junction solar cell. Therefore, a series of device physical characterizations were applied to understand the loss mechanism in our devices. According to the external quantum efficiency (EQE) spectra, the absorption edge of Se_0.7_Te_0.3_ solar cells is red shifted compared with pure Se solar cells (Fig. 3c). The full spectrum integral *J*_SC_ of Se_0.7_Te_0.3_ solar cells is 9.9 mA/cm^2^, close to the *J*_SC_ from the *J*–*V* curve. However, the collection efficiency of photogenerated carriers at long wavelengths is weak, which is always attributed to the short carrier diffusion length or nonradiative recombination centers in Se_0.7_Te_0.3_ absorber. The width of the depletion region (*x*_d_) of Se solar cells is 260 nm (Additional file 1: Fig. S15a) and the carrier diffusion length (*L*_d_) is 480 nm (Additional file 1: Fig. S15c). Thus, the optimal thickness of Se films is 740 nm, so the absorber should be thinner to reduce the carrier recombination loss. To explore the *V*_OC_ loss mechanism in the Se_0.7_Te_0.3_ solar cells, we conducted the device physical characterizations to analyze the recombination loss through *A* and the light intensity dependent *V*_OC_. The *A* (1–2) of the device implied that the main recombination mechanism in Se_0.7_Te_0.3_ solar cells is interface recombination. The *J–V* curves of the device were measured at different light intensities from 1 to 100 mW/cm^2^. Figure 3d shows that the *V*_OC_ and logarithm light intensity have a linear relationship in accordance with Eq. (3) [50].

3 $${V}_{\mathrm{OC}}=\frac{m{k}_{\mathrm{B}}T}{q}\mathrm{ln}I,$$

while the *J*_SC_ and light intensity satisfy the power law in accordance with Eq. (4) [50].

4 $${J}_{\mathrm{SC}}\propto {I}^{\alpha },$$

where *I*, *m*, *k*_B_, *q*, and *α* represent the light intensity, a constant, Boltzmann constant, elementary charge and logarithmic coefficient, respectively. The extracted *m* and *α* are 1.7 and 0.9, respectively. When *m* is larger than 1 and *α* is smaller than 1, the device performance is governed by the defect-related nonradiative recombination. *V*_OC_ deficit (defined by (*E*_g_ − *V*_OC_)/*q*) of Se_0.7_Te_0.3_ solar cell is 1.04 eV. It is known that the radiation recombination loss at room temperature is less than 0.3 V [51], much smaller than the real *V*_OC_ loss in our devices. Hence, the nonradiative recombination loss (1.04 − 0.3 = 0.74 eV) dominates 72% of total *V*_OC_ loss. To sum up, the performance of Se_0.7_Te_0.3_ device is governed by the ZnO/Se_0.7_Te_0.3_ interface recombination, and it can be minimized by interface energy band engineering or increasing the doping concentration of the absorber.

**Fig. 4:**
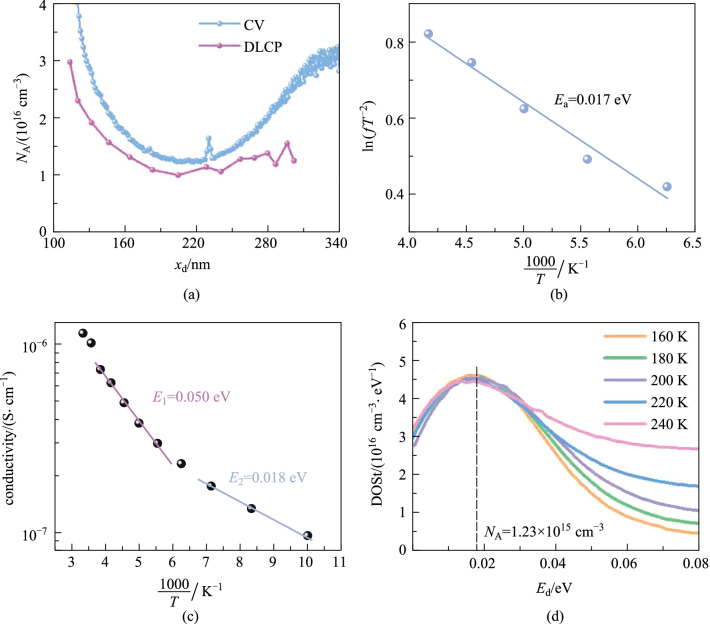
**a** Relationship between *N*_A_ and *x*_d_ measured by *C–V* and DLCP. **b** Arrhenius plot. **c** Temperature dependent dark-state conductivity. **d** Defect DOSt

Next, we further identified the interface defect information by *C–V*, DLCP and AS measurement. The *C–V* and DLCP curves are shown in Additional file 1: Fig. S16. To acquire the defect concentration, an abrupt heterojunction model was used to fit the experimental data. The capacitance and voltage satisfied the following relationship (Eq. (5)) [52].

5 $$\frac{1}{{C}^{2}}=\frac{2({V}_{\mathrm{bi}}-V)}{{A}^{2}\varepsilon q{N}_{\mathrm{A}}},$$

where *V*_bi_, *A*, *ε* and *N*_A_ represent for a built-in electric field, electrode area, permittivity and doping concentration, respectively. The intercept of the linear fitting (Additional file 1: Fig. S16a) on the *x*-axis represents the built-in potential (*V*_bi_ = 0.377 V), which is close to the *V*_OC_ of 0.348 V. The small *V*_bi_ results from the small Fermi energy level difference between ZnO (− 4.32 eV) and Se_0.7_Te_0.3_ (− 4.73 eV). Therefore, it is important to increase the free hole density of Se_0.7_Te_0.3_ in the future. The doping density calculated through *C–V* and DLCP measurement are *N*_A,CV_ = 1.65 × 10^16^ cm^−3^ and *N*_A,DLCP_ = 1.06 × 10^16^ cm^−3^, respectively. Interface defects can be calculated by the difference between *N*_A,DLCP_ and *N*_A,CV_ (Fig. 4a), the interface defect concentration of the device is 5.9 × 10^15^ cm^−3^, which acts as non-radiative recombination centers, and hence affects the charge extraction. Interfacial defects may derive from interfacial Se or Te vacancies and the ZnO/Se_1−*x*_Te_*x*_ lattice mismatch.

Temperature dependent AS measurement was further performed to study the defect depth and defect density of state (DOSt). According to the AS and differential capacitance spectra (Additional file 1: Fig. S17), there is a defect signal at the frequency range from 10^2^ to 10^4^ Hz and at the temperature range from 180 to 240 K. The defect depth (*E*_d_) can be calculated by Arrhenius formula (Eq. (6)) [53],

6 $$\mathrm{ln}\left(f{T}^{-2}\right)=-\frac{{E}_{\mathrm{d}}}{{k}_{\mathrm{B}}}\frac{1}{T}+\xi ,$$

where ƒ is the frequency, *ξ* is a constant without physical meaning. As shown in Fig. 4b, the fitted *E*_d_ is 0.017 eV. To further confirm defect depth obtained with AS, we measured the temperature dependent dark *I*–*V* curves from 80 to 320 K (Additional file 1: Fig. S18) and calculated defect depth *E*_a_ by Eq. (7) [54].

7 $$\sigma ={\sigma }_{0}\mathrm{exp}\left(-\frac{{E}_{\mathrm{a}}}{{k}_{\mathrm{B}}T}\right),$$

where *σ* means conductivity and *σ*_0_ is a constant without physical meaning. As shown in Fig. 4c, two shallow defect levels (0.050 and 0.018 eV) were observed. The shallower one was in line with *E*_d_ measured by AS, but the deeper one was not detected by AS. Combining the experimental results of temperature dependent AS and *I*–*V*, we inferred that there are two kinds of defect in the Se_0.7_Te_0.3_ films. Se (or Te) vacancy is easy to form, compared with the Se (or Te) interstitial defect, thus we assigned the 0.050 and 0.018 eV defects to Se and Te vacancy in Se_0.7_Te_0.3_ films, respectively.

The DOSt can be calculated by Eq. (8) [55],

8 $$\mathrm{DOSt }\left({E}_{\mathrm{d}}\right)=-\frac{{V}_{\mathrm{bi}}}{q{x}_{\mathrm{d}}}\frac{\mathrm{d}C}{\mathrm{d}f}\frac{f}{{k}_{\mathrm{B}}T},$$

where ƒ is the frequency and *x*_d_ is the width of depletion region. The DOSt of Se_0.7_Te_0.3_ film is shown in Fig. 4d. The concentration by integrating the defect DOSt was 1.23 × 10^15^ cm^−3^, which is two orders of magnitude higher than that of the traditional high-efficiency CdTe and Cu_2_(In,Ga)(S,Se)_2_ thin film solar cells [56]. More effort should be done to reduce the interface and bulk defects in the future.

## Conclusion

In conclusion, ZnO/Se_1−*x*_Te_*x*_ solar cells were fabricated in a full vacuum environment at low temperature (less than 200 °C). We found that the Zn^2+^ exposed surface of ZnO ETL would bond with Se to form a high-quality ZnO/Se_1−*x*_Te_*x*_ heterojunction interface during the post-annealing process. We then tuned the bandgaps of Se_1−*x*_Te_*x*_ to the optimal value of S–Q limit (1.36 eV) by alloying 30% Te with 70% Se. Consequently, a superior efficiency of 1.85% was achieved based on ITO/ZnO/Se_0.7_Te_0.3_/Au device. The analysis of the recombination mechanism of the Se_0.7_Te_0.3_ device implied that the defects of ZnO/Se_0.7_Te_0.3_ interface and Se_0.7_Te_0.3_ thin film may limit the device efficiency. Our results confirmed that the construction of efficient ZnO/Se_0.7_Te_0.3_ is feasible and represented an important advance for the realization of stable, efficient and green Se_1−*x*_Te_*x*_ solar cells.

## Supplementary Information

**Additional file 1: Figure S1.** Se_1−*x*_Te_*x*_ (*x*=0.2, 0.3, 0.4, 0.5 from left to right) blocks sintered. **Figure S2.** XRD scans of (a) unannealed and (b) annealed Se_1–*x*_Te_*x*_ thin films onto ZnO. It is notable that the (101) peaks appear randomly with *x*, which need further study. **Figure S3.** (a) Sample picture of Se_0.7_Te_0.3_ thin film annealed and SEM images of Se_0.7_Te_0.3_ thin film annealed at (b) 150 °C, (c) 200 °C, (d) 250 °C. **Figure S4.** EDS spectrum of Se_0.7_Te_0.3_ thin film annealed at 200 °C. **Figure S5.** AFM morphology of Se_1–*x*_Te_*x*_ films before anneal with (a) *x* = 0.2, (b) 0.3, (c) 0.4, (d) 0.5 thin film. AFM morphology of Se_1–*x*_Te_*x*_ films after 200 °C anneal with (e) x = 0.2, (f) 0.3, (g) 0.4, (h) 0.5. **Figure S6.** (a) Reflectivity and (b) transmissivity of Se_1–*x*_Te_*x*_ films. **Table S1.** The calculated ratio of Se_1–*x*_Te_*x*_ films by the Bragg's Law based on the (102) peak offsets from Se and Te standard card. The 2Theta of (102) peak of Se and Te is 43.694 and 38.252, respectively. The fitted FWHM of (102) peak in Se_1–*x*_Te_*x*_ films. **Table S2.** The p and RH of Se_1–*x*_Te_*x*_ film by Hall effect measurement. The carrier concentration of Se_0.8_Te_0.2_ film is too small to be measured. **Figure S7.** (a) UPS spectra and feature position determination, including the low and high energy cutoff of the crystalline Se_0.7_Te_0.3_ thin film. (b) Energy level diagram of Se_0.7_Te_0.3_. The detailed calculation process to obtain the VBM and CBM. **Table S3.** m ⊖ and m ⊖ of each substance in the reaction formula at room temperature. **Table S4.** The calculated △ m ⊖ and △ m ⊖ of reactions between Se and ZnO (TiO_2_) at 200 °C annealing temperature. **Figure S8.** (a) The images of annealed Se films deposited on ZnO and TiO_2_; (b) the efficiency statistics of Se solar cells with ZnO and TiO_2_ as ETL, respectively. **Figure S9.** The photographs of ZnO/Se (50 nm) sample (a) before annealing and (b) after annealing at 220 °C. (c) The photographs of ZnO sample. XPS of pure ZnO and ZnO/Se (thermally decomposed). (d) Se 3d, (e) Zn 2p. (f) The photographs of Se films after annealing on glass and glass/ZnO. **Figure S10.** (a) The fitted bandgap and (b) XRD scans of ZnO films by magnetron sputtering. **Figure S11.** AFM morphology with roughness parameter of ZnO films by magnetron sputtering. **Figure S12.** The J-V curves of (a) unaged and (b) one-month aged Se_1–*x*_Te_*x*_ (x = [Te] = 0.2, 0.3, 0.4, 0.5) devices. (c) The EQE and integrated JSC of one-month aged Se_1–*x*_Te_*x*_ devices. **Figure S13.** The efficiency statistics of (a) Se_1–*x*_Te_*x*_ (x = [Te] = 0.2, 0.3, 0.4, 0.5) solar cells and (b) Se_0.7_Te_0.3_ solar cells at different aging times. **Table S5.** Device performance parameters of Se_1–*x*_Te_*x*_ thin film solar cells after 1-month exposure to air, showing the better parameters than those of the newly fabricated devices as shown in TABLE II in the manuscript. **Figure S14.** Atomic structures of a monovacancy diffusing along the chain of Se_0.7_Te_0.3_. For clarity, we show only one layer.
