# Supplementary material for: Fabrication and characterization of ZnO/Se1-xTex solar cells
Source: Front Optoelectron. 2022 Sep 8;15(1):36. doi: 10.1007/s12200-022-00040-5 (PMC9756246; doi:10.1007/s12200-022-00040-5)
Supplement: Supplementary file 1 — Additional file 1: Figure S1. Se1−xTex (x=0.2, 0.3, 0.4, 0.5 from left to right) blocks sintered. Figure S2. XRD scans of (a) unannealed and (b) annealed Se1–xTex thin films onto ZnO. It is notable that the (101) peaks appear randomly with x, which need further study. Figure S3. (a) Sample picture of Se0.7Te0.3 thin film annealed and SEM images of Se0.7Te0.3 thin film annealed at (b) 150 °C, (c) 200 °C, (d) 250 °C. Figure S4. EDS spectrum of Se0.7Te0.3 thin film annealed at 200 °C. Figure S5. AFM morphology of Se1–xTex films before anneal with (a) x = 0.2, (b) 0.3, (c) 0.4, (d) 0.5 thin film. AFM morphology of Se1–xTex films after 200 °C anneal with (e) x = 0.2, (f) 0.3, (g) 0.4, (h) 0.5. Figure S6. (a) Reflectivity and (b) transmissivity of Se1–xTex films. Table S1. The calculated ratio of Se1–xTex films by the Bragg's Law based on the (102) peak offsets from Se and Te standard card. The 2Theta of (102) peak of Se and Te is 43.694 and 38.252, respectively. The fitted FWHM of (102) peak in Se1–xTex films. Table S2. The p and RH of Se1–xTex film by Hall effect measurement. The carrier concentration of Se0.8Te0.2 film is too small to be measured. Figure S7. (a) UPS spectra and feature position determination, including the low and high energy cutoff of the crystalline Se0.7Te0.3 thin film. (b) Energy level diagram of Se0.7Te0.3. The detailed calculation process to obtain the VBM and CBM. Table S3. m ⊖ and m ⊖ of each substance in the reaction formula at room temperature. Table S4. The calculated △ m ⊖ and △ m ⊖ of reactions between Se and ZnO (TiO2) at 200 °C annealing temperature. Figure S8. (a) The images of annealed Se films deposited on ZnO and TiO2; (b) the efficiency statistics of Se solar cells with ZnO and TiO2 as ETL, respectively. Figure S9. The photographs of ZnO/Se (50 nm) sample (a) before annealing and (b) after annealing at 220 °C. (c) The photographs of ZnO sample. XPS of pure ZnO and ZnO/Se (thermally decomposed). (d) Se 3d, (e) Zn 2p. (f) The photogra [file 12200_2022_40_MOESM1_ESM.pdf]

Supporting Information

Fabrication and characterization of $ZnO/Se_{1-x}Te_x$ solar cells

Jiajia Zheng, Liuchong Fu, Yuming He, Kanghua Li, Yue Lu, Jiayou Xue, Yuxuan Liu, Chong Dong, Chao Chen and Jiang Tang*

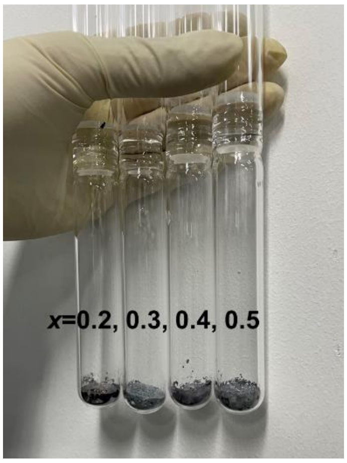

Figure S1. $Se_{1-x}Te_x$ (x=0.2, 0.3, 0.4, 0.5 from left to right) blocks sintered.

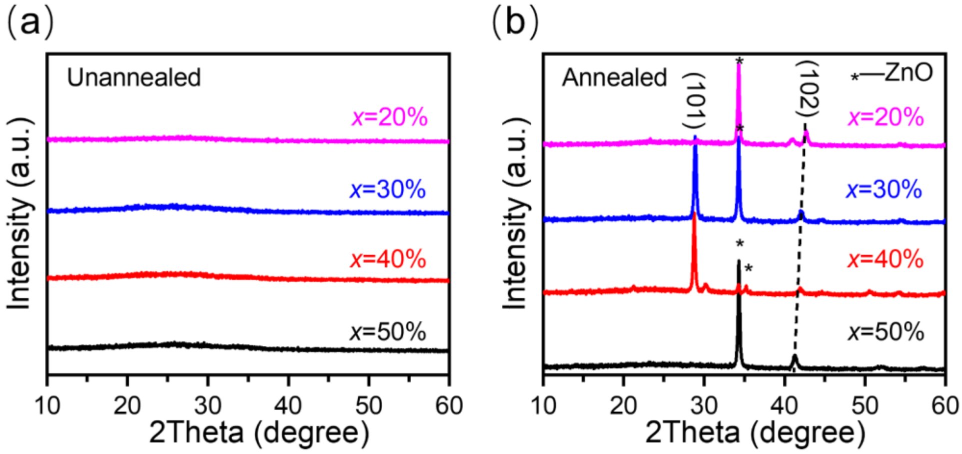

Figure S2. XRD scans of (a) unannealed and (b) annealed $Se_{1-x}Te_x$ thin films onto ZnO. It is notable that the (101) peaks appear randomly with x, which need further study.

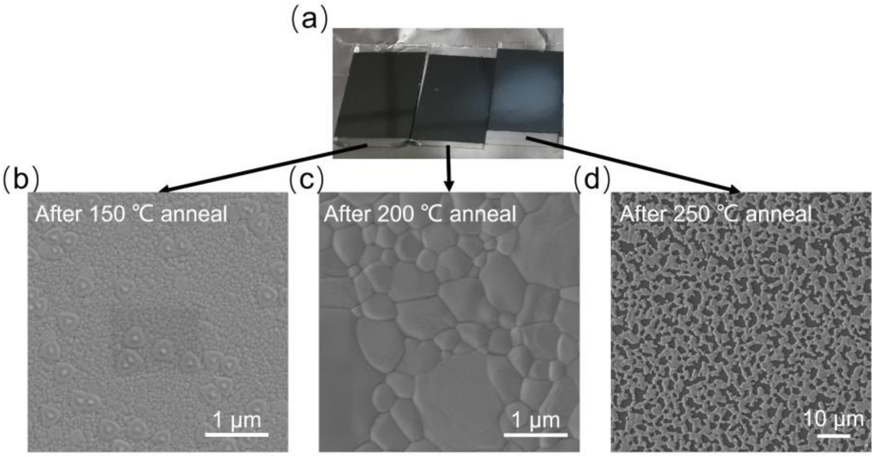

Figure S3. (a) Sample picture of $Se_{0.7}Te_{0.3}$ thin film annealed and SEM images of $Se_{0.7}Te_{0.3}$ thin film annealed at (b) 150 °C, (c) 200 °C, (d) 250 °C.

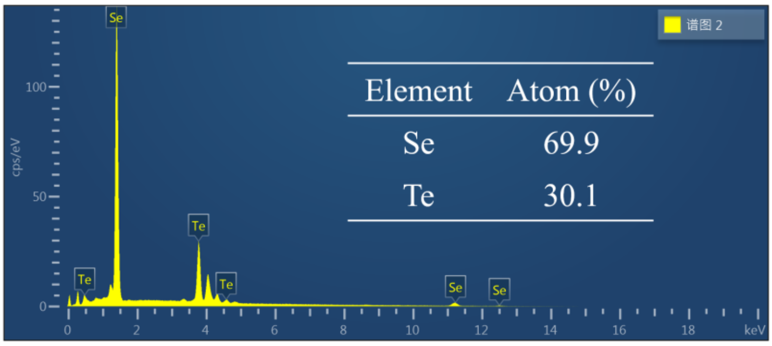

Figure S4. EDS spectrum of $Se_{0.7}Te_{0.3}$ thin film annealed at 200 °C.

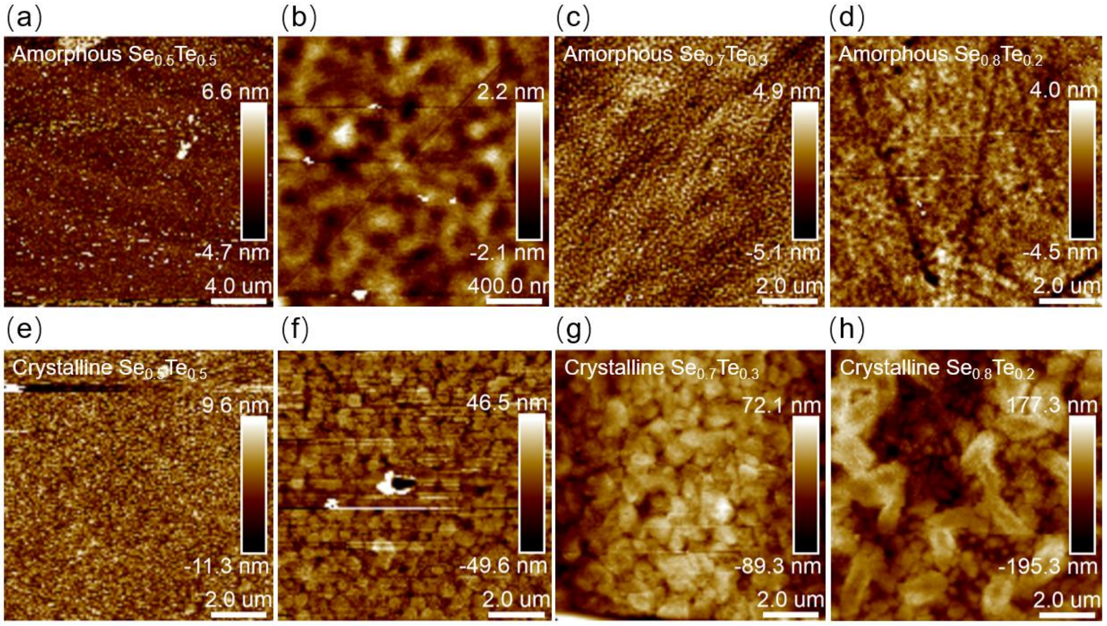

Figure S5. AFM morphology of $Se_{1-x}Te_x$ films before anneal with (a) x=0.2, (b) 0.3, (c) 0.4, (d) 0.5 thin film. AFM morphology of $Se_{1-x}Te_x$ films after 200 ℃ anneal with (e) x=0.2, (f) 0.3, (g) 0.4, (h) 0.5.

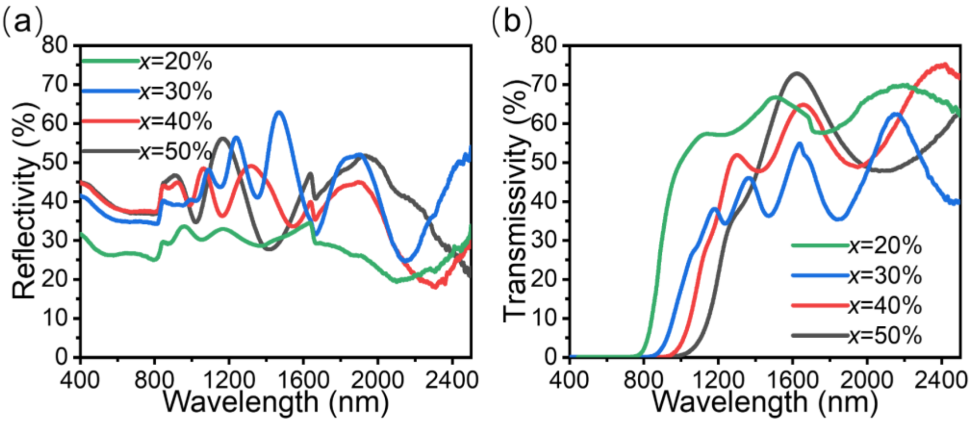

Figure S6. (a) Reflectivity and (b) transmissivity of $Se_{1-x}Te_x$ films.

Table S1. The calculated ratio of $Se_{1-x}Te_x$ films by the Bragg's Law based on the (102) peak offsets from Se and Te standard card. The 2Theta of (102) peak of Se and Te is 43.694 and 38.252, respectively. The fitted FWHM of (102) peak in $Se_{1-x}Te_x$ films.

	$Se_{0.8}Te_{0.2}$	$Se_{0.7}Te_{0.3}$	$Se_{0.6}Te_{0.4}$	$Se_{0.5}Te_{0.5}$
2Theta/degree	42.617	41.933	41.367	40.840
x	0.190	0.294	0.395	0.491
FWHM	0.473	0.479	0.569	0.589

Table S2. The p and R_H of $Se_{1-x}Te_x$ film by Hall effect measurement. The carrier concentration of $Se_{0.8}Te_{0.2}$ film is too small to be measured.

	$Se_{0.8}Te_{0.2}$	$Se_{0.7}Te_{0.3}$	$Se_{0.6}Te_{0.4}$	$Se_{0.5}Te_{0.5}$
p/cm^{-3}	N.A.	1.88×10^{14}	2.13×10^{15}	1.16×10^{16}
R_H	N.A.	3.32×10^{4}	2.93×10^{3}	5.40×10^{2}

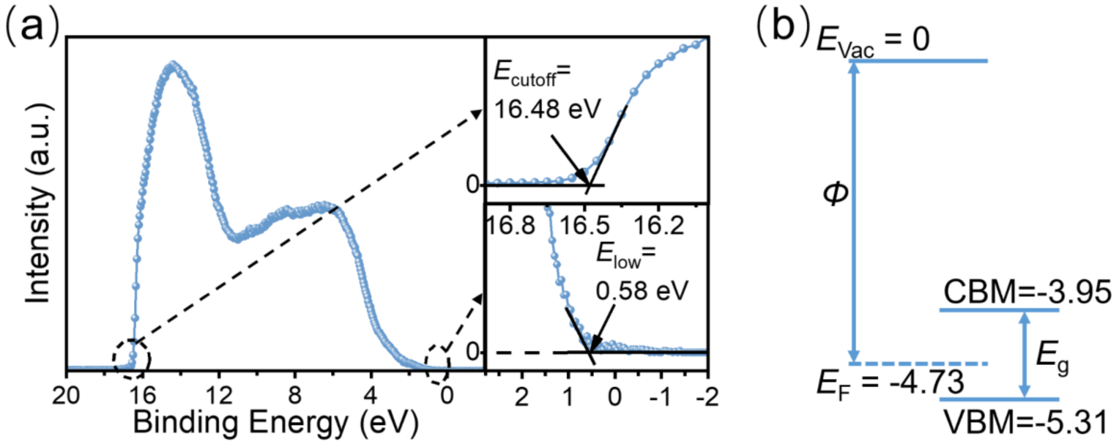

Figure S7. (a) UPS spectra and feature position determination, including the low and high energy cutoff of the crystalline $Se_{0.7}Te_{0.3}$ thin film. (b) Energy level diagram of $Se_{0.7}Te_{0.3}$. The detailed calculation process to obtain the VBM and CBM:

UPS test is based on Einstein photoelectric law, meeting the Equation (S1) as follows.[1]

$$E_{high}=h\nu-\phi \qquad \text{(S1)}$$

where $h\nu$ (equal to 21.21 eV) is photon energy excited by He I, E_{high} is high energy cutoff, ϕ is the work function of material. E_{high} (16.48 eV) is determined by linearly

extrapolating the high binding energy portion of the spectrum and finding the intercept with the background signal [Figure S7(a)]. Combining E_{high} and hv, we obtained the ϕ as 4.73 eV. According to energy band theory of semiconductors [Figure S7(b)], we can calculate the Fermi level (E_F) according to Equation (S2),

$$E_F = E_{vac} - \phi \tag{S2}$$

where E_{vac} (equal to 0) is the vacuum level. The E_F is -4.73 eV.

The difference between E_F and valence band maximum (VBM) is generally referred to the cutoff at its low binding energy (E_{low}) onset as described by Equation (S3).

$$\text{VBM} = E_F - E_{low} \tag{S3}$$

Combining the E_{low}=0.58 eV [Figure S7(a)], the VBM is calculated as -5.31 eV.

The difference between VBM and conduction band minimum (CBM) is equal to band gap (E_g) as addressed by Equation (S4).

$$\text{CBM} = \text{VBM} + E_g \tag{S4}$$

Combining the VBM (-5.31 eV) and E_g [1.36 eV, Figure 1(e) in the manuscript], the CBM of $Se_{1-x}Te_x$ is -3.95 eV.

Table S3. $H_m^{\ominus}$ and $S_m^{\ominus}$ of each substance in the reaction formula at room temperature.

Substance	$H_m^{\ominus}$ (kJ·mol^{-1})	$S_m^{\ominus}$/J·(℃·mol)$^{-1}$	Ref.
Se (g)	227.1	174.8	[2]
ZnO (s)	-350.46	43.65	[2]
ZnSe (s)	-163	84	[2]
SeO_2(s)	-225.4	67.49	[3]
TiO_2(s)	-944.08	50.62	[2]
$TiSe_2$(s)	-352.70	94.10	[4]

Table S4. The calculated $\triangle_r H_m^{\ominus}$ and $\triangle_r S_m^{\ominus}$ of reactions between Se and ZnO (TiO_2) at 200 ℃ annealing temperature.

Reaction formula	$\triangle_r H_m^{\ominus}$ (kJ·mol^{-1})	$\triangle_r H_m^{\ominus}$ (kJ ℃$^{-1}$·mol^{-1})
$3Se\ (g) + 2ZnO\ (s) \xrightarrow{200\ ℃} 2ZnSe\ (s) + SeO_2(s)$	-531.78	0.38
$3Se\ (g) + TiO_2\ (s) \xrightarrow{200\ ℃} 2TiSe_2\ (s) + SeO_2(s)$	138.88	-0.064

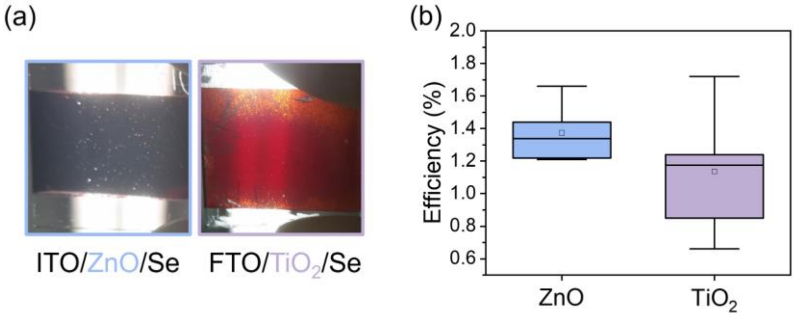

Figure S8. (a) The images of annealed Se films deposited on ZnO and TiO_2; (b) the efficiency statistics of Se solar cells with ZnO and TiO_2 as ETL, respectively.

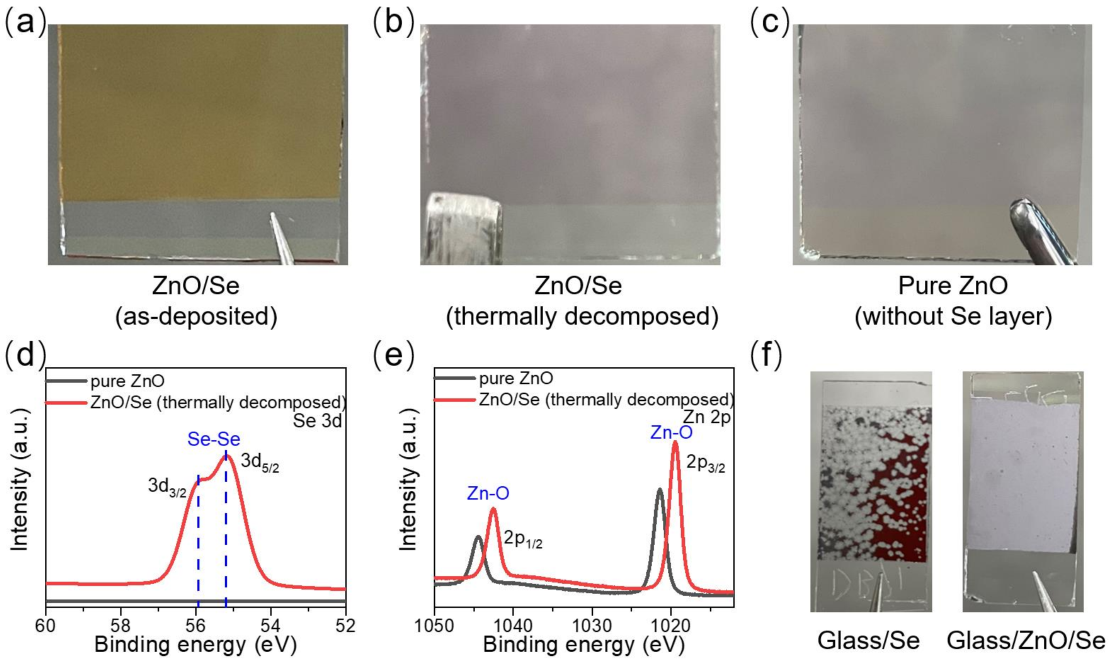

Figure S9. The photographs of ZnO/Se (50 nm) sample (a) before annealing and (b) after annealing at 220 ℃. (c) The photographs of ZnO sample. XPS of pure ZnO and ZnO/Se (thermally decomposed). (d) Se 3d, (e) Zn 2p. (f) The photographs of Se films after annealing on glass and glass/ZnO.

The experimental details to prove the existence of ZnSe layer and illustrate the effect of ZnSe:

50 nm thick Se films were evaporated on ZnO [Figure S8(a)], subsequently the ZnO/Se samples were annealed at 220 ºC for 1 min to thermally decompose the coated Se film completely [Figure S8(a-b)].

XPS measurement was conducted on ZnO/Se (thermally decomposed) film and pure ZnO film [Figure S8(c)] to analyze the presence of Zn-Se. The Se 3d peaks of ZnO/Se (thermally decomposed) film proved the existence of Se element [Figure S8(d)]. Compared with the pure ZnO film, Zn 2p peaks of ZnO/Se (thermally decomposed) film shift toward low binding energy, indicating the presence of Zn-Se (or Zn-Se-O) [Figure S8(e)].

When Se is deposited on the unreactive substrate [such as glass, Figure S8(f)], Se is easy to be thermally decomposed during the annealing process at 200 ºC due to the low melting point and high vapor pressure, which can lead do the large pinholes. As a comparison, when Se film was deposited on ZnO substrate and annealed at the same condition, the pinholes (or thermal decomposition) were not observed [Figure S8(f)]. Combining the above-mentioned proof about the existence of Zn-Se, we reasonably contribute the pinhole-free improvement to the interfacial strong bonding between Se and Zn. Overall, ZnSe can improve the adhesion and carrier transport between ZnO and Se or $Se_{1-x}Te_x$ layers, and thus is conducive to the performance of solar cells.

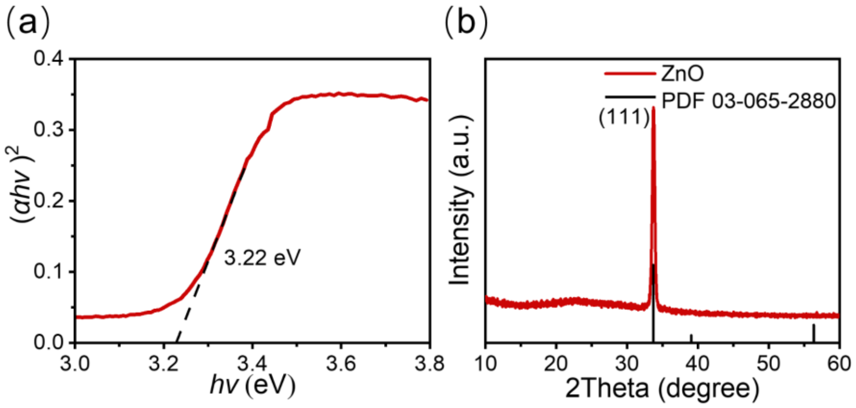

Figure S10. (a) The fitted bandgap and (b) XRD scans of ZnO films by magnetron sputtering.

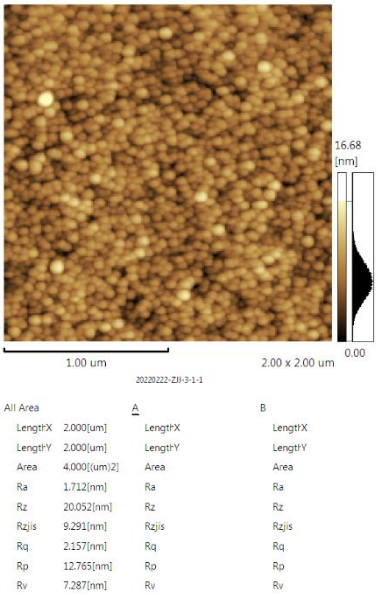

Figure S11. AFM morphology with roughness parameter of ZnO films by magnetron sputtering.

The detailed descriptions of ZnO on $Se_{0.7}Te_{0.3}$ devices:

The small roughness (1.712 nm) of ZnO means the smooth and uniform surface, which can be beneficial to form the uniform $Se_{1-x}Te_x$ films and good contact between ZnO and $Se_{1-x}Te_x$. The compact surface could effectively avoid the direct contact between ITO electrode and $Se_{1-x}Te_x$ films, therefore, it is beneficial to reduce the leakage current of the solar cells. Therefore, the smooth, uniform and compact ZnO is conducive for solar cell fabrication process.

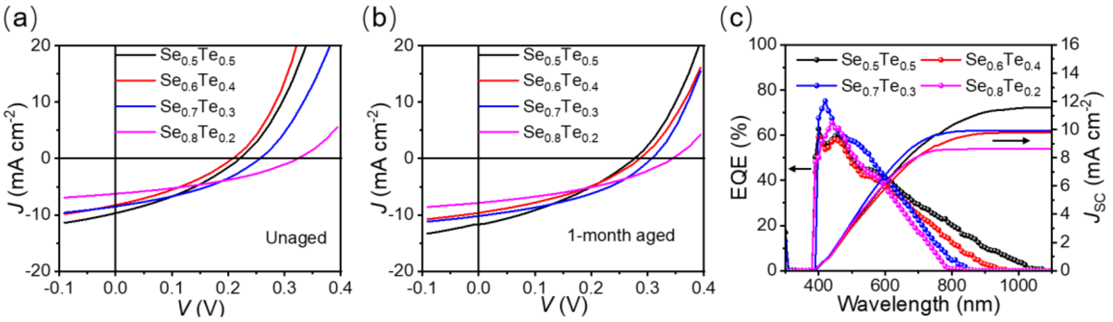

Figure S12. The *J*-*V* curves of (a) unaged and (b) one-month aged $Se_{1-x}Te_x$ (x=[Te]=0.2, 0.3, 0.4, 0.5) devices. (c) The EQE and integrated J_{SC} of one-month aged $Se_{1-x}Te_x$ devices.

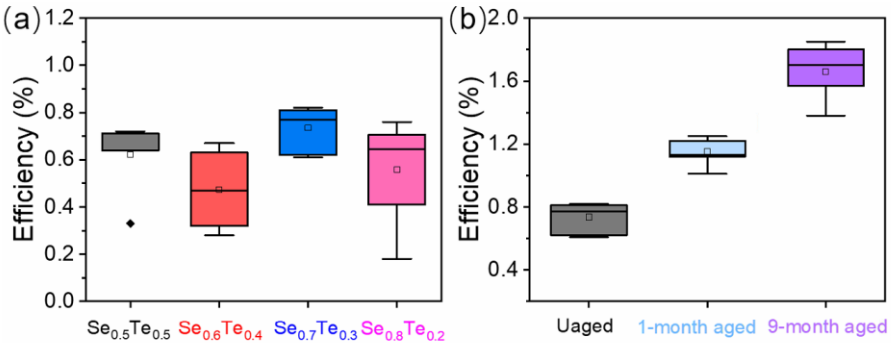

Figure S13. The efficiency statistics of (a) $Se_{1-x}Te_x$ (x=[Te]=0.2, 0.3, 0.4, 0.5) solar cells and (b) $Se_{0.7}Te_{0.3}$ solar cells at different aging times.

Table S5. Device performance parameters of $Se_{1-x}Te_x$ thin film solar cells after 1-month exposure to air, showing the better parameters than those of the newly fabricated devices as shown in TABLE II in the manuscript.

x	V_{OC} (V)	J_{SC} (mA cm^{-2})	FF (%)	PCE (%)	R_s (Ω)	R_{sh} (Ω)
0.2	0.343	7.9	38.0	1.02	200	1203
0.3	0.307	10.2	40.0	1.25	124	882
0.4	0.286	9.6	36.8	1.01	133	746
0.5	0.276	11.7	34.3	1.11	137	617

The analysis about A:

According to the single-diode model of thin-film solar cells, the J-V formula can be described by Equation (S5)[5],

$$J = J_0\left\{\exp\left[\frac{q(V-JR_s)}{AkT}\right]-1\right\}+\frac{V-JR_s}{R_{sh}}-J_{SC} \qquad \text{(S5)}$$

where J_0, q, V, R_s, A, k, T, R_{sh} and J_{SC} mean reverse saturation current density, elementary charge, voltage, series resistance, quality factor, Boltzmann constant, absolute temperature, shunt resistance and short circuit current density, respectively. Based on the Equation (S5), Prof. Sites from Colorado State University studied an efficient algorithm to extract the J_0, A, R_s and R_{sh},[6] and this approach was widely popularized.[7] The J-V data between the optimum voltage (V_{mp}) and V_{OC} were selected to fit the diode parameters.[8]

In general, the A of 1.34-1.41 is good enough for solar cell when the buffer layer is

heavily doped. The value of A can also be obtained from the carrier concentration of buffer and absorber layers [Equation (S6)][9],

$$A = 1 + \frac{\varepsilon_a N_{A,a}}{\varepsilon_b N_{D,b}} \tag{S6}$$

where ε_a, ε_b, $N_{A,a}$ and $N_{D,b}$ represent the permittivity of absorber and window layer, the carrier concentration of absorber and buffer layer, respectively.

Through Hall effect measurement, the carrier concentration after 1-month of aging is 1.88×10^{14} cm^{-3} ($N_{A,a}$), while the carrier concentration after 9-month of aging is too small to be measured. Therefore we chose $Se_{0.7}Te_{0.3}$ device after 1-month of aging to compare these two methods. The values of ε_a, ε_b and $N_{D,b}$ are 19, 10 and 6.2×10^{14} cm^{-3} (by Hall effect measurement), respectively. The calculated A of the 1-month aging device is 1.58, which is close to the value (1.56) by fitting the dark J-V curve. The 9-month device had lower $N_{A,a}$, thus its A is considered to be smaller, consistent with the fitted A of 1.34-1.41. The above results indicate that A is not only related to the interfacial defect concentration of PN junctions, but also to the carrier concentrations of buffer and absorber layers. In our work, A is small because the carrier concentration of the absorber ($Se_{0.7}Te_{0.3}$) is lower than that of the buffer layer (ZnO).

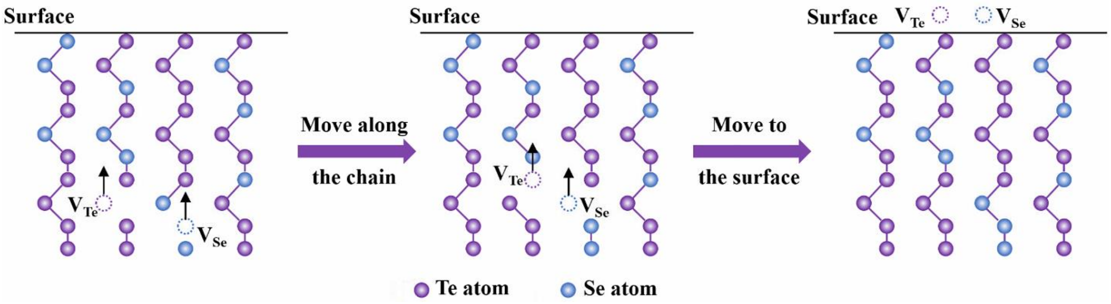

Figure S14. Atomic structures of a monovacancy diffusing along the chain of $Se_{0.7}Te_{0.3}$. For clarity, we show only one layer.

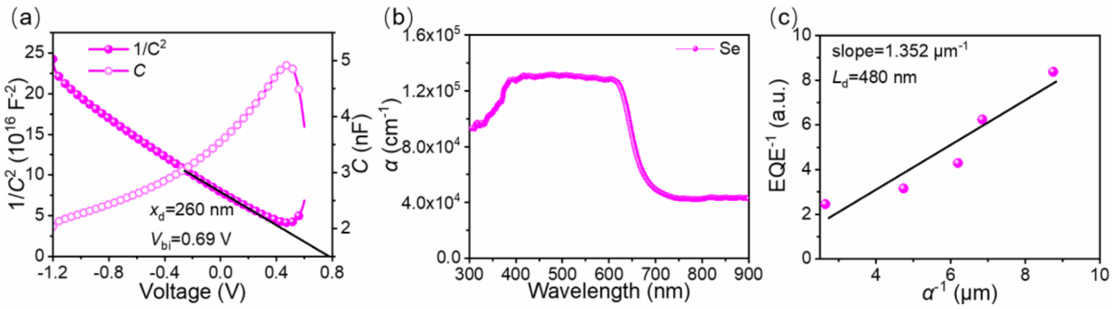

Figure S15. (a) The *C*-*V* curve of Se solar cell; (b) the absorption coefficient (~1×10^5 cm^{-1} at the wavelength of 300-650 nm) of Se film; (c) the calculated carriers diffusion length (480 nm) of Se solar cell.

The calculation processe of (L_d):

The L_d can be calculated based on the near-band edge EQE as shown in Figure 3c in the manuscript. In Se solar cells, the carrier generated by the near-band edge wavelength is far away from the space charge region, so it undergoes a long diffusion process before the collection. The carrier diffusion length calculation is based on the Equation (S7)[10]:

$$\mathrm{EQE}(\lambda)=1-\frac{\exp(-\alpha(\lambda)x_d)}{\alpha(\lambda)L_d+1} \quad \text{(S7)}$$

where $\alpha(\lambda)$ is the absorption coefficient as shown in Figure S15b, and x_d is the depletion zone width (260 nm) as shown in Figure S15a. Equation (S8) can be simplified according to the Taylor expansion because of the small $\alpha(\lambda)$ and x_d:

$$\mathrm{EQE}(\lambda)=\frac{L_d+x_d}{L_d+\frac{1}{\alpha(\lambda)}} \quad \text{(S8)}$$

$$\frac{1}{\mathrm{EQE}(\lambda)}=\frac{L_d}{L_d+x_d}+\frac{1}{L_d+x_d}\times\frac{1}{\alpha(\lambda)} \quad \text{(S9)}$$

Then, the L_d (480 nm) can be extrapolated from fitting the slope of $\mathrm{EQE}(\lambda)^{-1}$-$\alpha(\lambda)^{-1}$ ragnging from 650-700 nm as shown in Figure S15c according to Equation (S9).

As shown in Figure 3c in the manuscript, the near-band edge EQE of $Se_{0.7}Te_{0.3}$ solar cell drops slowly, therefore, it is not suitable for fitting L_d with the above methods.

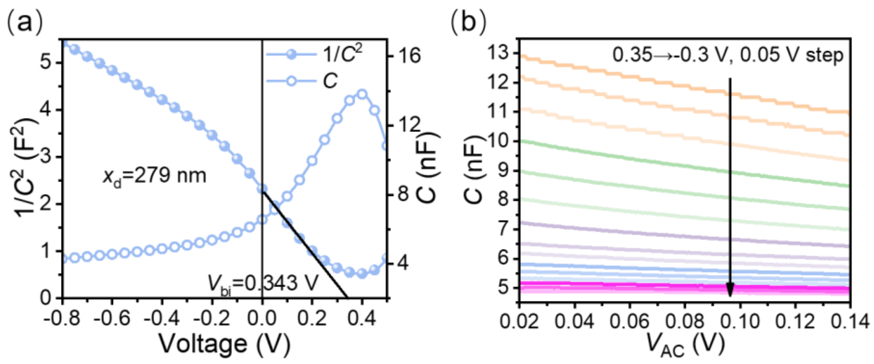

Figure S16. (a) The *C*-*V* curve and (b) DLCP results of $Se_{0.7}Te_{0.3}$ device.

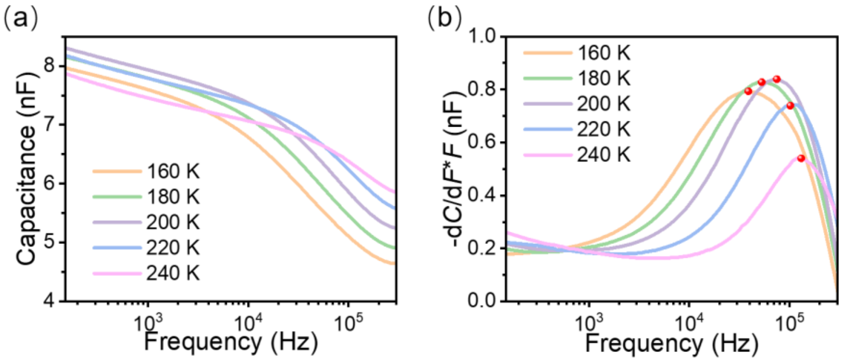

Figure S17. (a) AS and (b) differential AS of $Se_{0.7}Te_{0.3}$ device from 160 to 240 K.

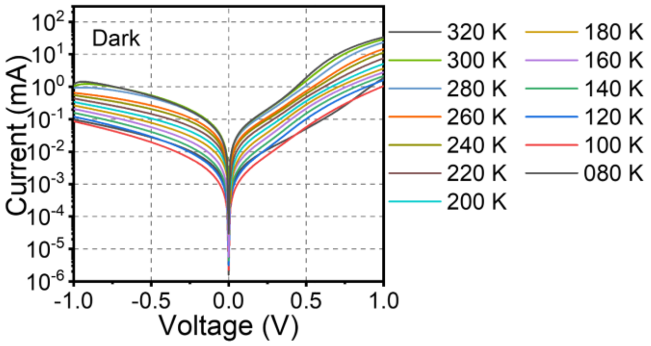

Figure S18. The dark *J*-*V* characterizations of ITO/ZnO/$Se_{0.7}Te_{0.3}$/Au device at various temperatures from 80 to 320 K.

References

[1] H. Zhu, A. Liu, Y. Y. Noh, *IEEE Electr. Device L.* **2019**, *40*, 769.

[2] J. A. Dean. *Lange's Handbook of Chemistry*, McGraw-Hill, New York **1999**.

[3] E. RosÉn. *Chemical Thermodynamics of Selenium*, Amster, **2005**.

[4] L. Zelenina, T. Chusova, A. Titov, *Russ. Chem. Bull.* **2011**, *60*, 581.

[5] S. S. Hegedus, W. N. Shafarman, *Prog. Photovoltaics* **2004**, *12*, 155.

[6] J. R. Sites, P. H. Mauk, *Solar cells* **1989**, *27*, 411.

[7] M. Luo, M. Leng, X. Liu, J. Chen, C. Chen, S. Qin, J. Tang, *Appl. Phys. Lett.* **2014**, *104*, 173904.

[8] J. R. Sites, *Sol. Energ. Mat. and Sol. C.* **2003**, *75*, 243.

[9] R. Scheer, H. W. Schock. *Chalcogenide Photovoltaics: Physics, Technologies, and Thin Film Devices*, Wiley-VCH, Baden-Württemberg, Germany **2011**.

[10] M. Stolterfoht, C. M. Wolff, J. A. Márquez, S. Zhang, C. J. Hages, D. Rothhardt, S. Albrecht, P. L. Burn, P. Meredith, T. Unold, *Nat. Energy* **2018**, *3*, 847.
